# Multilocus inference of species trees and DNA barcoding

**DOI:** 10.1098/rstb.2015.0335

**Published:** 2016-09-05

**Authors:** Diego Mallo, David Posada

**Affiliations:** Department of Biochemistry, Genetics and Immunology, University of Vigo, Vigo 36310, Spain

**Keywords:** species tree reconstruction, incomplete lineage sorting, multilocus barcoding, phylogenetic incongruence, multispecies coalescent, barcode gap

## Abstract

The unprecedented amount of data resulting from next-generation sequencing has opened a new era in phylogenetic estimation. Although large datasets should, in theory, increase phylogenetic resolution, massive, multilocus datasets have uncovered a great deal of phylogenetic incongruence among different genomic regions, due both to stochastic error and to the action of different evolutionary process such as incomplete lineage sorting, gene duplication and loss and horizontal gene transfer. This incongruence violates one of the fundamental assumptions of the DNA barcoding approach, which assumes that gene history and species history are identical. In this review, we explain some of the most important challenges we will have to face to reconstruct the history of species, and the advantages and disadvantages of different strategies for the phylogenetic analysis of multilocus data. In particular, we describe the evolutionary events that can generate species tree—gene tree discordance, compare the most popular methods for species tree reconstruction, highlight the challenges we need to face when using them and discuss their potential utility in barcoding. Current barcoding methods sacrifice a great amount of statistical power by only considering one locus, and a transition to multilocus barcodes would not only improve current barcoding methods, but also facilitate an eventual transition to species-tree-based barcoding strategies, which could better accommodate scenarios where the barcode gap is too small or inexistent.

This article is part of the themed issue ‘From DNA barcodes to biomes’.

## 1. Introduction

Gene trees based on single markers have been used as proxies for species phylogenies since the late 1970s. While the distinction between species and gene trees has been known for decades [1–3], the difficulty in obtaining multiple molecular markers delayed its explicit acknowledgement until very recently. The discordance between gene trees and species trees can be explained by both systematic—due to model misspecification—and stochastic—inherent to the finite amount of data and sampling process—error, but more importantly, this incongruence can also be the result of different evolutionary processes, mainly incomplete lineage sorting (ILS, table 1 collects all acronyms), gene duplication and loss (GDL) and horizontal gene transfer (HGT), but also hybrid speciation and gene flow [1–5]. Nowadays, advances in sequencing technologies have facilitated the acquisition of large multilocus datasets, unveiling extensive phylogenomic incongruence [6,7], and bringing back the species tree—gene tree dichotomy to the spotlight. In consequence, a plethora of species tree reconstruction methods have been developed in the last decade. While all of these methods aim for the same target, the species tree, they conform to a broad variety in terms of input data, model assumptions, estimation strategy and computational complexity. It is therefore important to take into account the characteristics of the data at hand in order to choose the most appropriate species tree methodology; or even better, to design the research project and the sequencing strategy taking into account the expected evolutionary processes involved and the most appropriate methods to analyse the data.

**Table 1.** Acronym table.

acronym	meaning
AFLP	amplified fragment length polymorphism
GDL	gene duplication and loss
GTP	gene tree parsimony
HGT	horizontal gene transfer
ILS	incomplete lineage sorting
MSC	multispecies coalescent
SNP	single nucleotide polymorphism

*DNA barcoding* consists of identifying the species at which a given sample pertains (either catalogued or new) and is usually carried out using a DNA sequence obtained from a single locus. These marker sequences or barcodes are not necessarily unique for a given species—because of intraspecific variability—and, therefore, most barcoding methods rely on the identifiability of two different ranges of variability, intraspecific and interspecific. This characteristic identifies the ‘barcode gap’, defined by the separation between the maximum within-species genetic distance and the minimum between-species genetic distance. Its existence is subject not only to genetic divergence among species, but also to the absence of deep coalescences (scenario where the most recent common ancestor for a given gene of all individuals from the same species precedes the speciation time) and gene flow, and, therefore, it is sensitive to the distinction between species and gene trees. Even in absence of these events, different clades can have different ranges of intraspecific and interspecific variability, cancelling the barcode gap when considering the reference tree as a whole. There are at least four different methodological strategies for species assignment using barcodes: tree-based, sequence-similarity-based, statistical and diagnostic methods [8]. *Tree-based* strategies use any classic phylogenetic method [9] to estimate the phylogeny (gene tree) of the reference barcodes together with the query sequence. The query is assigned to the species it clusters within. Therefore, these strategies rely on the barcode gap and assume that gene tree and species tree are topologically equivalent. *Sequence-similarity* methods look for the closest sequence among the references using similarity scores (e.g. BLAST [10]), assigning the species label of the closest reference to the query. Therefore, they also rely on the barcode gap because they assume that the intraspecific similarity is bigger than the interspecific. *Statistical* methods try to better exploit all the signals present in the data, accommodating uncertainty and yielding confidence measures of the assignment, at the expense of requiring extensive intraspecific sampling, population-size estimates and big computational efforts [11,12]. Finally, *diagnostic* methods analyse the reference looking for specific nucleotides that are able to assign potential queries to given species, neglecting the rest of the information [13,14]. Thus, they are less prone to be confounded by the absence of the barcode gap, whereas they strongly depend on the existence of a diagnostic combination of nucleotides.

Related to both species trees and DNA barcoding, *species delimitation* methods aim to determine the number of species present in a set of individual samples and their boundaries, and therefore generalizes the species assignment problem. Most single-locus species-delimitation methods rely on the distinction of the intra- and interspecific ranges of variability, and therefore are affected by the absence of the barcode gap in a similar manner to DNA barcoding. There are at least four different methodological strategies for species delimitation: genetic-distance-based, phylogenetic-based, divergence-based and allelic-exclusivity-based (reviewed in [15]). *Genetic-distance*-based methods directly rely on the barcode gap to delimitate species, using a user-specified fixed threshold of genetic distance (e.g. jMOTU [16]) or estimating it (e.g. ABGD [17]). *Phylogenetic-based* methods are based on the phylogenetic species concept (reviewed in [18]) and therefore rely on modelling two evolutionary branching patterns—intra- and interspecific—and detecting the transition between them. GMYC [19] and related methods model speciations under a Yule model and intraspecific variation with a coalescent process, whereas PTP [20] models two different Poisson branching processes (avoiding the need of ultrametric trees). The *divergence-based* method *K*/*θ* [21] looks for clades that diverged significatively more than expected by genetic drift, identifying them as different species. It uses both a sequence-distance matrix and a phylogenetic tree to compare the mean sequence diversity among clades (*K*) and the population mutation rate (*θ*), and it is intended for asexual organisms. *Allelic-exclusivity****-***based methods (e.g. Haplowebs [22]) look for clusters of individuals that share alleles that are mutually exclusive with other individuals, gathering the information from the co-occurrence of haplotypes in heterozygous individuals.

In this paper, we review the evolutionary events that generate species tree—gene tree discordance, methods for species tree reconstruction, the challenges we face when using them and their potential role in barcoding.

## 2. Species trees, population trees and gene trees

Species trees, the focus of this review, depict the evolutionary history of the sampled organisms. The nodes of a species tree represent speciation events, whereas the branches reflect the population history between speciations. The width of a branch in a species tree represents the effective population size (*N*_e_), whereas its lengths represent time, usually in years or number of generations. Population trees are similar to species trees, but consider the history of conspecific populations. Finally, gene trees represent the evolutionary history of the sampled gene copies. The nodes of a gene tree indicate coalescent events, which correspond, looking forward in time, to the process of DNA replication and divergence. Coalescent events can occur right before the speciation time, well before (deep coalescence) or right afterwards (gene flow). The length of the branches in a gene tree usually represents the amount of substitutions per site. Importantly, tree-based barcoding refers to the use of gene trees.

## 3. Evolutionary processes that generate species tree/gene tree discordance

**Figure 1.**
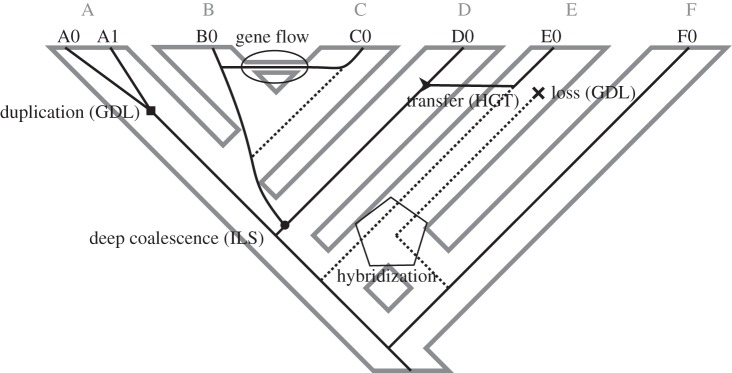
Evolutionary processes that generate species tree/gene tree incongruence. The figure shows the species tree (grey tree in the background) and a gene tree (black tree) tracking the evolutionary history of six species (A, B, C, D, E and F) and nine gene copies (A0α, A0β, B0, C0, C1, D0, E0, E1 and F0) in eight individuals (A0, B0, C0, C1, D0, E0, E1, F0). Each evolutionary process is indicated by a label and a specific figure in the node where it is mapped (duplication, square; loss, cross; transfer, arrow; deep coalescence, circle; hybridization, pentagon; gene flow, ellipse). Dashed lines indicate superfluous lineages that do not reach the present due to gene loss.

In spite of being conceptually different, species and gene trees are expected to be topologically equivalent under many evolutionary scenarios. Nevertheless, certain evolutionary processes disrupt this equivalence, decoupling their histories (figure 1).

### (a) Incomplete lineage sorting

**Figure 2.**
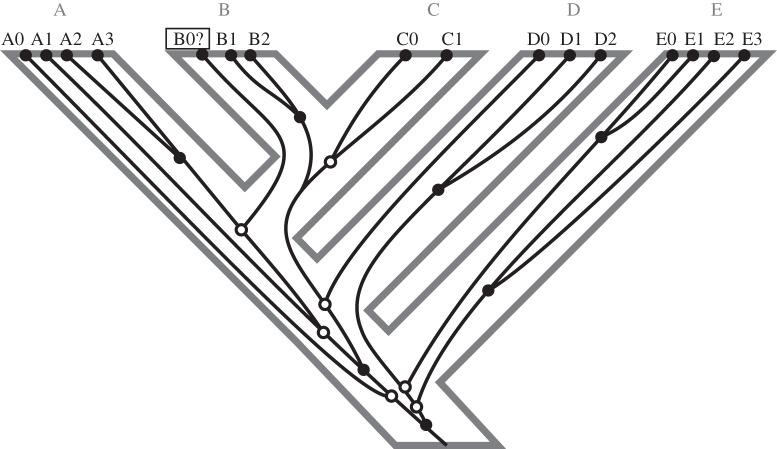
Multispecies coalescent model. The figure shows the species tree (grey tree in the background) and a gene tree (black tree) tracking the evolutionary history of five species (A, B, C, D and E) and several individuals per species. Each species tree branch corresponds to an independent coalescent process. Gene tree nodes are depicted with circles, where open circles indicate deep coalescences. The confounding effect of ILS on standard barcoding techniques is reflected here, for example between species A and B. The individual B0 from species B clusters with individuals A2 and A3 from species A therefore shows the absence of a barcode gap.

ILS, also known as deep coalescence or ancestral polymorphism, is the result of the retention of a genetic polymorphism along several speciation events. The posterior sorting of polymorphic lineages can make gene and species trees incongruent. Therefore, ILS is a special case of the consideration of how alleles evolve and sort within populations. ILS is usually modelled using the multispecies coalescent (MSC) model [23] (figure 2), which expands coalescent theory [24] to be applied on species trees. The discordance due to ILS increases with effective population size and decreases with species tree branch length. In consequence, ILS is mostly associated with closely related species, although it is not exclusive of them, as short branches can also occur deeper in time. Because of this, ILS is probably the most relevant source, together with hybrid speciation and gene flow, of gene tree—species tree incongruence for DNA barcoding.

### (b) Gene duplication and loss

GDL describes the copy of a locus into a different genomic location and its loss, the primary source of new genetic material driving the evolution of gene families [25]. GDL is the result of several known molecular mechanisms such as unequal crossing-over and retroposition [26]. Traditionally, before phylogenetic estimation, duplicated gene copies—and, therefore, the signature of GDL—are removed from the data in order to only consider orthologous gene copies (orthology prediction methods are reviewed in [27,28]). Most species tree reconstruction methods considering GDL follow a gene tree parsimony (GTP) approach [29] in which the parsimony score for a species tree is the minimum number of duplications that this implies given a collection of gene trees.

### (c) Horizontal gene transfer

HGT or lateral gene transfer corresponds to the integration in the genome of a portion of genetic material coming from a different species in a non-sexual fashion, thus disrupting species boundaries and vertical inheritance. This evolutionary process is widespread in non-eukaryotic organisms [30], although it is not restricted to them [31,32]. HGT can be modelled as a Poisson-distributed series of events, but most species tree methods considering it are based on GTP. As in the case of GDL, the signature of HGT is often detected and removed from the data based on phylogenetic incongruence, patchy distribution (presence or absence patterns) or compositional anomalies [30].

### (d) Hybrid speciation

Hybrid speciation corresponds to a speciation through interbreeding between members of two different species. The new species is therefore originated from two ancestral species, generating a reticulated history or species network. The new species may have the same number of chromosomes as its parent species (homoploid hybridization) or their sum (polyploid hybridization) [33]. This evolutionary process is fairly common in plants, but not restricted to them [34]. Although this process can mislead DNA barcoding [35], we are not aware of specific DNA barcoding strategies to tackle it.

### (e) Gene flow

Gene flow is the acquisition of genetic material through interbreeding across species boundaries. Unlike HGT, during gene flow, full genomes are transferred from one species to another via sex. Afterwards, introgressed genomes can be broken up by recombination, and different loci can follow alternative histories, eventually getting fixed in the new species or drifting away. Population trees are strongly affected by gene flow, whereas in species trees (as long as the biological species concept holds), gene flow can only occur during speciation or immediately after, a process represented by the isolation with migration model (IM) [36]. Gene flow is currently neglected by species tree reconstruction methods.

## 4. Species tree reconstruction methods

There is a broad variety of species tree reconstruction methods (table 2) that follow different methodological approaches in terms of evolutionary model, input data and computational requirements, making it difficult to choose a single criterion to arrange them into categories. Here, we classify them considering their input data, because the data determine most of their main assumptions and basic characteristics.

**Table 2.** Species tree reconstruction programs. For each program (the list is not exhaustive), the table indicates the evolutionary processes that generate species tree/gene tree discordance explicitly taken into consideration by the model (Ev. process; ILS, incomplete lineage sorting; GDL, gene duplication and losses, HGT, horizontal gene transfer), input data (MSAs, multiple sequence alignments; SNPs, single nucleotide polymorphisms), output data and the amount of data each software is intended to handle (scalability).

	Ev. process	strategy	input	output	scalability
ASTRAL I/II [37,38]	none	algorithm: quartet compatibility	unrooted trees	unrooted supertree	genome-wide
BUCKy [39,40]	none/ILS	Bayesian inference: concordance factors	unrooted distributions	unrooted species tree and gene tree distributions	multilocus
RF supertrees [41]	none	heuristic: distance	rooted trees	rooted supertree	genome-wide
MulRF [42]	none	heuristic: distance	unrooted trees	unrooted supertree	genome-wide
iGTP [43]	ILS or GDL	heuristic: reconciliation cost	rooted or unrooted trees	rooted or unrooted supertrees	genome-wide
SPRSupertrees [44]	HGT	heuristic: distance	unrooted trees	unrooted or rooted supertrees	genome-wide
GLASS, SD, MAC, STEAC, STAR [45–48]	ILS	algorithm: distance	rooted trees	rooted species tree	genome-wide
NJst/ASTRID [49,50]	ILS	algorithm: distance	unrooted trees	unrooted species tree	genome-wide
STEM [51]	ILS	algorithm: distance + likelihood	rooted trees, theta, rate	rooted species tree	genome-wide
MP-EST [52]	ILS	heuristic: pseudo-likelihood	rooted trees	rooted species tree	genome-wide
STELLS [53]	ILS	heuristic: pseudo-likelihood	rooted trees	rooted species tree	genome-wide
Guenomu [54]	ILS + GDL + HGT + distances	Bayesian inference: distance-based model	unrooted tree distributions	rooted species tree and unrooted gene tree distributions	genome-wide
PHYLDOG [55]	GDL	heuristic: likelihood	MSAs	rooted supertree	genome-wide
BEST [56]	ILS	Bayesian inference: MSC	MSAs	rooted species and gene tree distributions	small multilocus datasets
*BEAST [57]	ILS	Bayesian inference: MSC	MSAs	rooted species and gene tree distributions	small multilocus datasets
SVDQuartets [58,59]	ILS	algorithm: singular value decomposition of site pattern frequency matrix + quartet tree reconstruction	SNPs	unrooted species tree	genome-wide
Phylonet [60–62]	ILS + hybridization	heuristic (multiple): reconciliation cost/pseudo-likelihood/likelihood	unrooted gene trees	unrooted species networks	multilocus datasets
SNAPP [63]	ILS	Bayesian inference: MSC	SNPs	rooted species tree distribution	genome-wide

### (a) Supermatrix (concatenation)

The supermatrix or concatenation approach relies on joining all single-locus alignments into a multilocus alignment, which is used as input data for a standard phylogenetic estimation methodology (e.g. maximum-parsimony, maximum-likelihood, Bayesian inference and distance methods). The underlying assumption is that either all gene trees share the species history or the discordant phylogenetic signals cancel out when all the histories are considered together. If any of these assumptions holds, then the concatenated tree should be a reasonable proxy of the species tree phylogeny.

### (b) Supertree

The supertree approach consists of two steps. First, gene trees are estimated independently with any standard phylogenetic reconstruction method. Second, the resulting gene trees are combined into a single species tree or supertree. Most species tree reconstruction methods are supertree methods, although they can follow completely different strategies.

#### (i) Disagreement reduction

These methods do not model any evolutionary process. Instead, they try to find the supertree(s) that minimize(s) the disagreement among gene trees. This category includes consensus [64] and concordance methods such as BUCKy [39,40], ASTRAL [37] and ASTRAL-II [38]. Consensus methods build a tree with compatible gene tree bipartitions weighted by their frequencies while BUCKy does so using concordance factors. ASTRAL and ASTRAL-II maximize the number of quartets induced by the input gene trees. Matrix representation using parsimony [65,66] or likelihood [67] summarize gene tree topologies into a matrix representing the absence/presence of given nodes across the gene trees, which is then used to reconstruct the species tree under the corresponding optimality criterion. Finally, other methods try to minimize topological distances among gene trees, such as the RF [41,42] and MulRF [42] supertree approaches.

#### (ii) Single evolutionary process

Many species tree reconstruction methods explicitly consider a single evolutionary process. Some rely on the optimization of a gene tree–species tree reconciliation cost (GTP; [29]). These methods compute the number of deep coalescences, GDLs or HGTs necessary to explain the gene tree–species tree discordance, returning the species tree that minimizes them. The iGTP program [43] implements the reconciliation models for either ILS or GDL, whereas SPRSupertrees [44] does the same for HGT. Other types of methods that are focused on ILS calculate distance trees using coalescent times as speciation upper bounds, as orthologous gene copies in different species that obligatorily had to diverge before the speciation event. Here, we can include programs such as GLASS [45], STEAC [46], SD [47], MAC [48], STAR [46], NJst [49] or ASTRID [50] (most of them reviewed in [68]). STEM [51] algorithmically estimates the GLASS species tree under a likelihood framework. Finally, other methods also based on the MSC model use fast heuristic optimization procedures on a likelihood-like function in order to find the most likely species tree, such as MP-EST [52] and STELLS [53].

#### (iii) Multiple evolutionary processes

A few species tree reconstruction methods can consider multiple evolutionary processes at once. Models considering ILS and hybridization have been implemented in the program Phylonet, which can reconstruct species networks under parsimony [60], maximum-likelihood [61] and pseudo-likelihood [62] criteria. De Oliveira Martins *et al*. [54] proposed a Bayesian supertree method—implemented in the program Guenomu—that considers ILS, GDL, HGT and gene tree—species tree discordance. This method is based on a hierarchical Bayesian model, and calculates the posterior probability of the species tree given the gene trees upon several reconciliation costs and distances. This program takes as input posterior gene tree distributions estimated by any Bayesian gene tree estimation software (e.g. MrBayes; [69]).

### (c) Full data

A small family of species tree methods directly analyse the sequence data, thus using all the available information contained in the individual alignments.

#### (i) Modelling incomplete lineage sorting

SVDquartets [58,59] estimates the best topology for quartets of taxa based on the singular value decomposition of a matrix of site-pattern frequencies. Subsequently, the reconstructed quartets are assembled into a species tree using, for example, a tool such as Quartet MaxCut [70]. The SVDquartets method has been intended for single nucleotide polymorphism (SNP) data, but simulation studies suggest that it can perform well with multilocus datasets. Other ILS-aware methods use full probabilistic approaches in a Bayesian framework. Thus, SNAPP [63]—implemented in BEAST2 [71]—estimates species trees, divergence times and population sizes on SNP or amplified fragment-length polymorphism (AFLP) data, integrating over all possible gene (SNP/AFLP) trees (thus not estimating them). BEST [56] and *BEAST [57] implement an MSC model in order to co-estimate gene and species trees from sequence data, providing estimates of not only distributions of gene trees and species trees, but also of other important parameters such as population sizes under complex population dynamics [72,73] and divergence times using relaxed-clock models [74,75].

#### (ii) Modelling gene duplication and loss

PHYLDOG [55] relies on a birth–death probabilistic approach to jointly reconstruct species and gene trees from multiple gene family alignments.

## 5. Species tree accuracy

Most species tree reconstruction methods rely either directly or indirectly on estimated gene trees. Therefore, every condition able to mislead gene tree reconstruction will, to a greater or lesser extent, also affect final species tree accuracy. Bayzid & Warnow [76] conducted a simulation study showing a great correlation between gene tree and species tree accuracies, claiming that the advantage of the most accurate species tree reconstruction method in their experiments, *BEAST, was due to estimating much better gene trees. Therefore, different factors that affect the accuracy of gene tree estimation can also influence the accuracy of the resulting species trees.

Gene tree reconstruction methods are considered robust to missing data as long as the amount of phylogenetic signal is enough to obtain a reliable tree [77,78]. In fact, including taxa with a lot of missing data can improve the overall phylogenetic accuracy [79]. Nevertheless, new discussions on this topic have arisen recently [80–82]. When considering the species tree reconstruction step, we add one layer of complexity, because different genes can cover different taxa (incomplete taxon coverage). This situation can generate indecisive scenarios [83] characterized by extensive tree terraces that complicate phylogenetic analysis [84]. Very recently, Xi *et al*. [85] showed that at least concatenation and supertree methods (disagreement-based and ILS-based) are robust to random missing data provided a sufficiently large dataset, whereas non-randomly distributed missing data become more problematic [86]. Thus, concatenation is misled by non-randomly distributed missing data in combination with substitution-rate heterogeneity, and even worse with additional high levels of ILS. Supertree methods respond in different ways. Disagreement-based methods (ASTRAL and MRP at least) and MP-EST are quite robust to missing data, whereas STAR (and potentially other distance-based ILS supertree methods) is strongly misled by it.

Intralocus recombination splits genes into regions with different evolutionary histories, misleading gene tree estimation at different levels [87,88]. Nevertheless, according to Lanier & Knowles [89], species tree reconstruction methods—at least STEM—are robust to the effect of intralocus recombination. Moreover, in their simulations, the confounding effect of recombination was reduced by adding loci and/or individuals per species.

Conversely, gene flow can be an important misleading force for species tree estimation, depending on the migration model. Eckert & Carstens [90] showed that supertree ILS-based methods are robust to historical gene flow models (parapatric and allopatric), whereas the concatenation approach is not. Nevertheless, their results suggest that stepping-stone and, more importantly, n-island models of gene flow can strongly mislead supertree and concatenation approaches. Leaché *et al*. [91] further studied the effect of gene flow on both ILS-based supertree and full probabilistic Bayesian methods, showing that gene flow between sister species increases species tree topological accuracy, whereas gene flow between non-sister species strongly bias species tree estimation. Moreover, gene flow induces over-compression (species tree-branch length underestimation) and dilatation (population-size overestimation) to a different extent depending on the exact gene-flow model assumed.

The amount of HGT, GDL and ILS affects species tree accuracy even when these processes are explicitly considered by the model. While the accuracy of ILS-based methods decays with the amount of ILS [47,76,92], both high and low GDL or HGT rates mislead the inference of species trees [93]. In spite of not being explicitly considered, moderate levels of ILS do not worsen by much the accuracy of PHYLDOG's species trees, although they induce an overestimation of the number of duplications and losses [55]. Randomly distributed HGT does not dramatically decrease the accuracy of ILS-based fully probabilistic methods, although its accuracy drops when HGT is focused on a specific species tree branch [94]. The relative robustness under low and moderate levels of random HGT is also shared with quartet-based disagreement-reduction supertree methods—ASTRAL-II and wQMC [95]—concatenation and ILS-based supertree methods (NJst), whereas under high levels of HGT, quartet-based methods stand out in terms of accuracy (especially ASTRAL-II) [96].

The supermatrix approach is the most accurate species tree reconstruction method when the effect of ILS or HGT is low and/or the amount of phylogenetic signals per loci is small (e.g. short sequences) [97,98]. This advantage is due to the reduction of the noise/signal ratio by considering together all the phylogenetic information. The accuracy of non-supermatrix approaches is strongly depleted by loci with low phylogenetic signal (usually short genes) due to increased gene tree error. Several related strategies based on combining groups of loci to generate so-called supergenes have been proposed in order to diminish this issue. These solutions constitute a compromise between concatenation and supertree methods that try to improve the noise/signal ratio for each supergene without assuming that gene and species trees are topologically equivalent. The latest of these methods—weighted statistical binning [97]—has shown interesting improvements on the accuracy of different species tree reconstruction methods.

Full probabilistic species tree reconstruction methods stand out as the most accurate in most benchmarks that take them into consideration [49,55,76,99]. Nevertheless, these types of methods are only suitable for small datasets because of computational constraints. Among faster alternatives considering ILS, ASTRAL II and NJst/ASTRID are usually the most accurate [38,50,100]. MP-EST shows also very good performance in computer simulations, and in spite of being slower, is probably the most popular species tree method nowadays [101–104]. The program Guenomu is so far the only one capable of taking into account ILS, GDL and HGT simultaneously—using a non-parametric model—avoiding the need for an orthology-assignment step.

## 6. Multilocus species-delimitation methods

Multilocus species-delimitation methods share most models and strategies with species-tree reconstruction methods, but extend them in order to estimate the number of species, species assignment and species boundaries in the sample. These methods also take into consideration the species tree–gene tree dichotomy, usually relying on the MSC model to deal with ILS. Some species delimitation methods co-estimate both the species tree and the species delimitation, whereas others need pre-estimated species trees as input. Species delimitation methods are very relevant to DNA barcoding, because they could be used as a basal framework to develop new DNA barcoding strategies or could be directly applied to that purpose.

Several multilocus species-delimitation approaches have been proposed in recent years [15,105,106]. According to their input data, they conform to either the supertree or the full-data approach. At least three recently published methods pertain to the former. O'Meara [107] developed a GTP-based species-delimitation strategy that minimizes both gene tree conflict in interspecific regions (calculating a gene duplication cost) and excess of structure in within-species regions (using a cost of excess of triplet-overlapping, calculated using coalescent simulations). Ence & Carstens developed a multilocus species-delimitation method (SpedeSTEM [108]) that uses STEM to calculate the likelihood of alternative-delimitation hypothesis (species trees)—generated by hierarchical permutation of putative intraspecific groups—which are afterwards evaluated using information theory statistics such as the Akaike information criterion [109]. KC delimitation [107] is another ILS-based species-delimitation method, which estimates the species tree and delimitation that maximize gene tree probability under the simulations using MSC.

The remaining methods use sequence alignments as input, taking advantage of the full data in Bayesian full-probabilistic approaches. Grummer *et al*. [110] and Leaché *et al*. [111] proposed the use of a model-selection strategy (Bayes factors [112]) to select the best-fit species assignment based on the comparison of marginal likelihoods. The species assignments are proposed by the user, and the marginal likelihoods estimated using *BEAST or SNAPP, respectively. BPP [113–115] expands the strategy used by *BEAST to carry out species delimitation, and is capable of co-estimating both the species tree and the species delimitation or any of them given the other. This method explicitly explores the delimitation space by considering different combinations of pre-specified populations as the candidate species using a reversible jump Markov chain Monte Carlo (rjMCMC). BPP has been expanded recently (iBPP [116]) in order to consider not only molecular data, but also phenotypic traits. Finally, DISSECT [117] avoids the usage of the rjMCMC—which is computationally expensive—by considering each individual as a single species and modifying the node height prior to estimate branch/node collapsibility in *BEAST.

## 7. Species trees and barcoding

Barcoding methods try to identify the species at which a given DNA sample pertains, and therefore are related to species trees by the very nature of its purpose. Nevertheless, for practical reasons, the species tree–gene tree dilemma has been so far neglected, and most barcoding methods are based on a single locus. Nevertheless, the species tree–gene tree incongruence directly disturbs barcoding by modifying the extent of the barcode gap across the tree of life, getting even to vanishing it in certain clades. In the light of this problem and the latest advances on species tree reconstruction and multilocus species delimitation methods, Dowton *et al.* [118] encouraged to extend the current barcoding framework. Thus, they proposed a multilocus alternative based on the MSC model, which relies on *BEAST for the species tree estimation and on BPP for the subsequent species-delimitation step. Nonetheless, Collins & Cruickshank [119] demonstrated that for the data used by Dowton *et al.*, appropriately adjusting a classical method for the existing barcode gap is enough to equate the accuracies of the two frameworks. In light of these results, the authors discouraged the adoption of MSC-based multilocus methods until the current framework is comprehensively shown not to work, arguing that the new alternative is too costly in terms of computation and sequencing. Collins & Cruickshank argued that focusing on comprehensive sampling, complete reference libraries and developing further single locus methods would improve DNA barcode identification success in a more extensive way, avoiding the need for re-sequencing and curating new reference genes. Nevertheless, Yang & Rannala [120] very recently conducted a simulation study in which single-threshold barcode methods performed poorly, being largely outcompeted by BPP. They also show that the increase in sequencing costs of the proposed framework shift would not be so dramatic, because BPP obtains reasonable results even with a single locus; and 10 loci are enough to get high accuracy and precision. While BPP is computationally intensive, Yang & Rannala propose to alleviate the computational burden by reducing the size of the problem, analysing divergent groups of species as separate datasets. New alternative single-locus barcoding methods have also arisen recently, based on different strategies such as coalescent theory [11,12], machine learning [121], neural networks [122], fuzzy-set theory [123] and character-based logic [124]. Among them excel character-based barcoding methods, which are more accurate than the classical tree and similarity barcoding methods in scenarios with recent speciations (including situations in which a barcoding gap does not exist) [8] and therefore could constitute an appropriate compromise between classical and MSC-based barcoding. Nevertheless, in spite of being more robust to a barcoding overlap, character-based methods still require groups of nucleotides with diagnostic power, which may simply not exist for certain species for a given locus due to a relatively small sequence length or to a low substitution rate.

Current barcoding methods sacrifice a great amount of statistical power by considering only one locus, and the transition to multilocus barcodes might not be that expensive, because sample collection, DNA isolation and (partially) PCR would not require a large additional investment [125]. Most current single-locus barcoding strategies would benefit from the addition of extra loci: multilocus character-based methods could increase their accuracy by adding more diagnostic characters; statistical barcoding methods would gain additional power from the use of multiple, independent evidence [125]; and tree-based methods could use concatenated loci to improve phylogenetic accuracy for clades with poor phylogenetic signal. Moreover, the use of multiple loci would facilitate the transition to species tree-based strategies, which accommodate better possible barcoding overlaps. While full-probabilistic MSC-based barcoding approaches such as the one proposed by Dowton *et al*. [118] might be too computationally intensive—although they could become feasible by using a small number of loci and analysing well-diverged groups separately, future strategies could extend the current tree-based barcoding framework by using any of the fastest (but still accurate) species tree reconstruction methods reviewed in this paper (e.g. ASTRAL II, MP-EST or ASTRID) on multilocus barcodes, extending the current tree-based barcoding strategy to a species tree-based barcoding framework.
